# Supplementary material for: The Mind Under Pressure: What Roles Does Education Play in the Relationship Between Chronic Stress and Cognitive Ability?
Source: J Intell. 2025 Jan 23;13(2):13. doi: 10.3390/jintelligence13020013 (PMC11856239; doi:10.3390/jintelligence13020013)
Supplement: Supplementary file 1 [file jintelligence-13-00013-s001.zip › jintelligence-3353847-supplementary.pdf]

Supplementary Materials

The Mind Under Pressure: What Roles Does Education Play in the Relationship Between Chronic Stress and Cognitive Ability?

Online supplement

Table of Content

Table S1 *Categorical Demographic Variables by Educational Group* 2

Table S2 *Wording and Item Descriptives of the Standard Stress Scale (Gross and Seebaß 2016)* 3

Table S3 *Absolute and Relative Frequencies of the Reported Degrees* 4

Table S4 *Descriptive Statistics of Chronic Stress, Reasoning, and Vocabulary Separated by Level of Education* 5

Figure S1 *Descriptive Statistics of Chronic Stress, Reasoning, and Vocabulary Separated by Level of Education* 6

Figure S2 *Demo Item of the Reasoning Test (Fluid Cognitive Ability)*... 6

Table S5 *Regression Weights of the Control Variables Included* 7

Table S6 *Sensitivity Analysis with Complete Cases Only* 7

Table S7 *Measurement Invariance Checks for Matched Samples* 7

Table S8 *Comparison of Latent Covariances for Matched Samples* 8

Table S1

Categorical Demographic Variables by Educational Group

		Elementary (N = 401)		Secondary (N = 4,253)		Tertiary (N = 4,629)	
		N	%	N	%	N	%
Gender	male	128	31.92	1970	46.32	2472	53.40
	female	273	68.08	2283	53.68	2157	46.60
	NA	0	0.00	0	0.00	0	0.00
Retirement Status	not retired	313	78.05	3515	82.65	3947	85.27
	retired	88	21.95	738	17.35	682	14.73
	NA	0	0.00	0	0.00	0	0.00
Marital Status	married	237	59.10	2807	66.00	3132	67.66
	not married	41	10.22	488	11.47	617	13.33
	NA	123	30.67	958	22.53	880	19.01
Language	German	231	57.61	2596	61.04	2971	64.18
	Other	170	42.39	1657	38.96	1658	35.82
	NA	0	0.00	0	0.00	0	0.00
Disability	recognized	79	19.70	649	15.26	537	11.60
	none	321	80.05	3601	84.67	4092	88.40
	NA	1	0.25	3	0.07	0	0.00
ADHD Diagnosis	documented	12	2.99	53	1.25	41	0.89
	none	327	81.55	3676	86.43	4135	89.33
	NA	62	15.46	524	12.32	453	9.79

Table S2

Wording and Item Descriptives of the Standard Stress Scale (Gross and Seebaß 2016)

Item	Facet	*N*	*M*	*SD*	min	max
If I do not enjoy doing something, I usually do not have to do it.[a]	Work	9,333	2.74	1.10	1	5
If I do not take care of things by myself, nobody handles it.	Work	9,341	3.19	1.01	1	5
I am doing meaningful tasks. [a]	Work	9,335	4.00	0.82	1	5
I often feel lonely.	Social	9,344	1.79	0.90	1	5
My performance is appreciated adequately. [a]	Social	9,328	3.55	0.88	1	5
There are people I can count on. [a]	Social	9,344	4.47	0.69	1	5
Usually I have a restorative sleep.	Exhaustion	9,341	3.59	1.12	1	5
I often think about problems.	Exhaustion	9,345	3.27	1.00	1	5
After a normal day I am exhausted.	Exhaustion	9,342	3.10	1.07	1	5
I am afraid about what my life will be like in three years.	Future	9,338	1.97	0.99	1	5
I am looking forward to my future. [a]	Future	9,325	3.88	0.91	1	5

Note. [a] reversely phrased items: items were recoded before aggregation; here the original (i.e., not recoded) values are reported.

Table S3

Absolute and Relative Frequencies of the Reported Degrees

Category	Content	Group	*N*	%
0	[0A/1A] No qualification	excluded	61	.01
1	[2B] School-leaving qualification; vocational preparation	elementary	215	.02
2	[2A] Intermediate school-leaving qualification	elementary	186	.02
3	[3A] Entry qualification for universities of applied sciences (higher education entrance qualification)	secondary	226	.02
4	[3B] Apprenticeship, vocational school, school of public health (basic professional skills)	secondary	3,325	.32
5	[3C] Civil servant clerical class	secondary	75	.01
6	[4A] Entry qualification for universities of applied science (higher education entrance qualification) (second cycle)	secondary	123	.01
7	[4B] Apprenticeship, vocational school, school of public health (basic professional skills) (second cycle)	secondary	505	.05
8	[5B] Diploma from a college of public administration or school of public health; other qualifications from a civil servant executive class	tertiary	1,917	.18
9	[5A] Bachelor, Master, Diploma/Magister, State Examination	tertiary	2,561	.25
10	[6] Doctorate, habilitation	tertiary	151	.01
NA	no information available	excluded	1,072	.10

Note. The categories are based on the ISCED-97 classification scheme.

Table S4

Descriptive Statistics of Chronic Stress, Reasoning, and Vocabulary Separated by Level of Education

	Elementary (N = 401)			Secondary (N = 4,253)			Tertiary (N = 4,629)		
	N	*M*	*SD*	*N*	*M*	*SD*	*N*	*M*	*SD*
Demographics									
Age	401	52.72	11.48	4,253	52.12	10.35	4,629	50.89	11.04
Income	283	1,593.67	834.00	3,219	1,904.92	829.32	3,510	2,472.71	1,018.12
Chronic Stress									
Sum Score	394	28.76	5.60	4,216	27.38	5.17	4,606	26.61	4.83
Work	401	2.92	0.61	4,253	2.84	0.55	4,629	2.78	0.55
Social	401	2.07	0.70	4,252	1.97	0.60	4,629	1.87	0.55
Exhaustion	401	3.06	0.85	4,252	2.92	0.78	4,629	2.91	0.72
Future	400	2.32	0.91	4,252	2.11	0.84	4,626	1.96	0.76
Reasoning									
Sum Score	317	6.41	3.10	3,528	7.75	2.77	3,845	8.92	2.46
Parcel 1	317	0.59	0.31	3,524	0.68	0.27	3,843	0.77	0.24
Parcel 2	315	0.50	0.33	3,517	0.63	0.29	3,828	0.73	0.25
Parcel 3	315	0.58	0.33	3,505	0.69	0.30	3,822	0.78	0.26
Vocabulary									
Sum Score	318	66.21	12.31	3,539	72.17	9.13	3,853	76.60	7.74
Parcel 1	318	0.86	0.15	3,537	0.91	0.10	3,853	0.94	0.08
Parcel 2	318	0.84	0.13	3,536	0.90	0.09	3,852	0.92	0.08
Parcel 3	318	0.70	0.18	3,536	0.77	0.15	3,852	0.84	0.13
Parcel 4	318	0.69	0.14	3,537	0.74	0.12	3,852	0.81	0.11
Parcel 5	318	0.80	0.15	3,538	0.84	0.11	3,853	0.88	0.10

Figure S1

Descriptive Statistics of Chronic Stress, Reasoning, and Vocabulary Separated by Level of Education

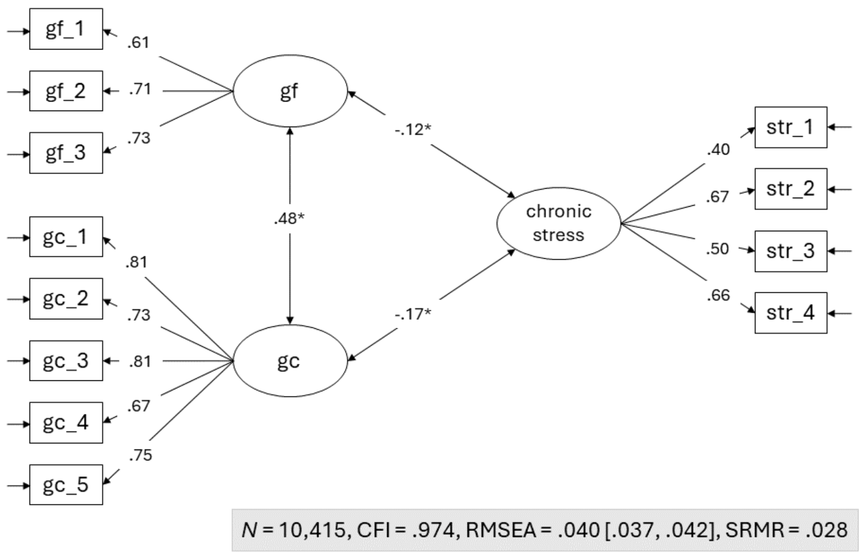

Figure S2

Demo Item of the Reasoning Test (Fluid Cognitive Ability)

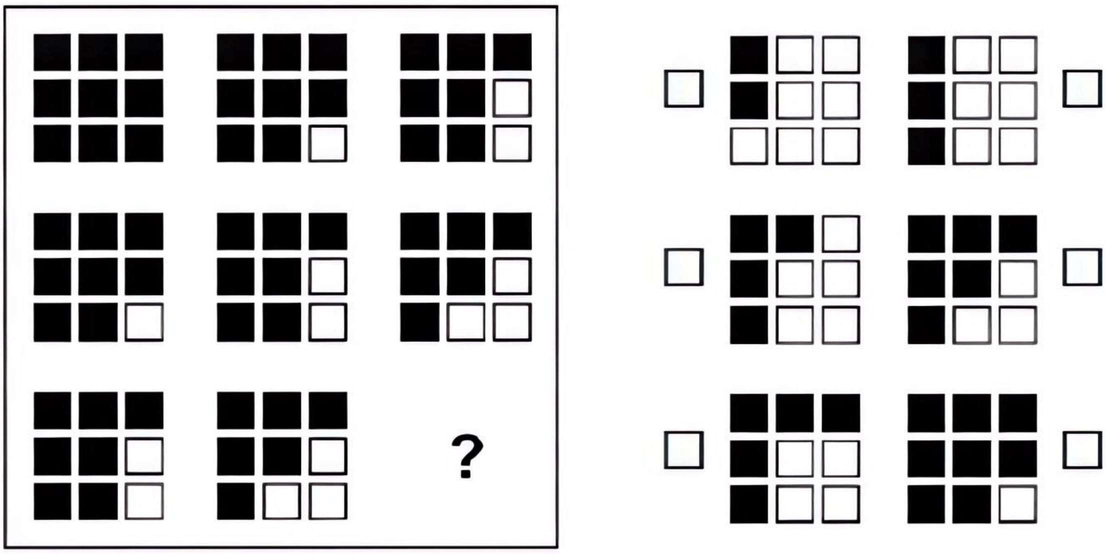

Note. The test began with two demo items, which were used by the interviewer to explain the procedure to the participants. The total time limit was set liberally at an average of 9 minutes. The test was administered on a Tablet PC. There was a total of 12 items in three increasingly difficult blocks of four items each.

Table S5

Regression Weights of the Control Variables Included

	Stress		gc		gf	
	est.	p	est.	p	est.	p
Age	.061	<.001	.091	<.001	-.401	<.001
Gender	-.067	<.001	.027	.013	.096	<.001
Income	-.294	<.001	.285	<.001	.254	<.001
Retirement	-.109	<.001	-.062	<.001	-.099	<.001
Language	-.017	.178	.126	<.001	.077	<.001
Marital Status	-.078	<.001	.002	.860	.035	.002

Table S6

Sensitivity Analysis with Complete Cases Only

Model	N	CFI	RMSEA	95% CI	SRMR
Basic Model (BM)	7,758	.974	.042	[.039;.045]	.026
BM + Control Variables	7,758	.969	.046	[.045;.048]	.032
BM + Control Variables + Fixed Correlations	7,758	.969	.046	[.044;.048]	.032

Table S7

Measurement Invariance Checks for Matched Samples

		CFI	ΔCFI	RMSEA	ΔRMSEA	SRMR	ΔSRMR
elementary vs. secondary	Configural	.972		.039		.039	
	Metric	.973	<.001	.037	<.001	.039	<.001
	Scalar	.974	<.001	.036	<.001	.040	.001
	Fixed Covariances	.971	.003	.037	.001	.048	.008
elementary vs. tertiary	Configural	.971		.041		.043	
	Metric	.971	<.001	.041	<.001	.045	.002
	Scalar	.967	.004	.043	.002	.047	.002
	Fixed Covariances	.963	.004	.045	.002	.060	.013

Note. Samples were matched using the k-nearest neighbor algorithm, implemented in the *MatchIt* Package in R (Ho et al. 2011). As the implemented algorithm allows for dichotomous treatment variables only, we report two separate analyses.

Table S8

Comparison of Latent Covariances for Matched Samples

	elementary vs. secondary				elementary vs. tertiary			
	elementary		secondary		elementary		tertiary	
	est.	p	est.	p	est.	p	est.	p
gf / gc	.583	<. 001	.492	<.001	.586	<.001	.469	<.001
gf / stress	-.152	.104	.084	.337	-.163	.081	-.082	.393
gc / stress	-.055	.487	-.054	.465	-.062	.438	.008	.916
